# Genetic diversity and genome-wide associations for frost survival in sorghum

**DOI:** 10.1186/s12870-025-07014-7

**Published:** 2025-07-28

**Authors:** Natalja Kravcov, Luisa Neitzert, Rod Snowdon, Steffen Windpassinger

**Affiliations:** Department of Plant Breeding, Justus Liebig University, Giessen, Germany

**Keywords:** Abiotic stress, Climate adaptation, Freezing, Genetic diversity, GWAS, Juvenile Frost tolerance, *S. bicolor*

## Abstract

**Background:**

The ability to withstand periods of light frost during juvenile development is an important adaptive trait for sorghum (*Sorghum bicolor* L. Moench) production in high-latitude temperate areas. Frost tolerance would allow for earlier sowing and prolongation of growing season and maturity, thereby increasing yield potential. Using a broad diversity set (*n* = 285), a field cold emergence trial and two semi-controlled frost survival experiments were conducted to investigate the phenotypic diversity and genetic architecture of frost survival traits.

**Results:**

The results show high phenotypic diversity for frost survival, including superior genotypes combining high frost tolerance and cold emergence levels via quantitative trait inheritance. Tolerance to temperatures above and below 0 °C was not correlated, suggesting separate genetic control. The found QTL do not overlap for the traits of frost survival and plant emergence under cold sowing conditions. Genome-wide association studies revealed four significant associations for frost survival, where the QTLs are located on chromosomes Sb02, Sb07, and Sb08. For the trait of emergence under cold sowing conditions, a total of 12 QTLs were found on chromosomes Sb01, Sb03, Sb04, Sb05, and Sb06.

**Conclusion:**

A good overview regarding the suitability of the tested genotypes for early sowing was obtained due to the large diversity investigated in this study, with surprisingly high survival rates of numerous genotypes after frost stress. These results will contribute to the breeding of cultivars with improved cold and frost tolerance, facilitating better adaptation for production in high-latitude temperate areas, as well as earlier sowing to avoid summer drought.

**Supplementary Information:**

The online version contains supplementary material available at 10.1186/s12870-025-07014-7.

## Background

In times of climate change, new or alternative crops are especially important to improve climate resilience and biodiversity in temperate areas. One option in this regard is sorghum (*Sorghum bicolor* L. Moench, 2n = 20), which is currently the fifth most important crop worldwide [1–3]. Sorghum is well adapted to drought and low input conditions due to its high water and nutrient efficiency [4]. The use of sorghum is diverse and ranges from food to animal feed to biogas substrate. In Central and Northern Europe, its implementation as a novel crop is obstructed mainly by its sensitivity to cold temperatures. As a C_4_ plant with a tropical background, sorghum generally prefers temperatures above 15 °C [5, 6]. Temperatures in many agricultural production areas are increasing during springtime due to climate change, but are still below the optimum temperature for early sowing of sorghum. Short frost periods even in mid to late spring are not uncommon in Central Europe and can completely kill sorghum plants. Presently, the only option to avoid cold stress at the juvenile stage is late sowing, but this shortens the remaining growing season, limiting yield potential and increasing the risk of reproductive cold stress due to delayed flowering [7, 8]. Earlier sowing would also help to avoid heat and drought stress during critical developmental stages in summer. Cold-tolerant sorghum forms, such as *kaoliangs* from China, are available, but they exhibit negative agronomic characters including low grain yield and tall plant height [9]. Some better-performing genotypes with moderate cold adaptation are available, but these genotypes can only tolerate temperatures above freezing [10]. For this reason, a distinction is made between cold tolerance (0 °C to 15 °C) and frost tolerance (below 0 °C).

Cold stress has different effects during juvenile stage on plant emergence, biomass development, physiological and biochemical pathways [11–14]. Among other things, it impairs various stages of photosynthesis and transpiration [15, 16] and can cause molecular and metabolic changes [17]. Root development can also be impaired by cold stress [18, 19]. Furthermore, traits such as emergence in cold soils and early vigor are strongly influenced by seed characters. Quantitative trait locus (QTL) analyses have shown that relevant traits such as germination, emergence and vigor under cold conditions are quantitatively controlled by multiple QTL [6, 16, 20, 21]. The involvement of multiple gene loci with minor effects on juvenile cold tolerance represents a challenge for marker-assisted selection. However, progress has already been made in the molecular breeding of complex traits using population genomics and high-throughput phenotyping. This methodology has also been used to improve the cold tolerance of elite varieties by crossing in tolerance alleles from Chinese accessions [22]. Cold tolerance shows a strong interaction between genotype and environment, which makes general predictions difficult [12, 13]. Some QTL (on chromosomes Sb02, Sb04, Sb07 and Sb09) for cold tolerance are located closely to gene loci for the important agronomic traits, tannin content (*Tan*1, *Tan*2) and plant height (*Dw*1, *Dw*3). Consequently, a possible cause for the loss of cold tolerance in US grain sorghums could be years of selection for short, tannin-free genotypes, although, QTL for cold tolerance that are not linked to these traits have also been discovered [23].

Cold tolerance during juvenile development (related to temperatures above freezing) is already well studied in sorghum [5, 11, 20], but seedling frost tolerance (related to temperatures below 0 °C) has not yet been reported well. To our best knowledge, only one recent study [24] has analyzed its genetic architecture on RIL populations, but no GWAS on a broad diversity set has been published yet. Further, no information on accessions with high frost tolerance which would be useful for tolerance breeding are available.

In temperate latitudes in Europe, short frost periods can occur even after sowing in late spring, potentially leading to total crop failure for farmers due to killing of young sorghum plants. This forces farmers to sow later, which shortens the available maturation time and reduces yield potential. Temperatures below 0 °C lead to changes in cell structures, which is one reason why cold-tolerant genotypes may still be susceptible to frost [25]. Therefore, juvenile frost tolerance is a crucial adaptation trait required for earlier sowing dates in temperate latitudes.

Surviving short periods of frost depends on the ability to protect the tissue and photosynthetic apparatus from reactive oxygen species (ROS). ROS also have a gene-regulating function and are important in the response to stress in plants, including cold or frost. Low concentrations can help prevent cell damage, but high concentrations lead to cell death [26, 27]. Plants, including sorghum are able to reduce ROS under abiotic stress with increased ROS-detoxifying enzymes [28, 29]. Cell damage at temperatures below 0 °C can be caused not only by ice formation in the tissue, but also by dehydration of cells. As a result, proteins and molecules are altered, causing membrane damage [25, 30]. Flavonoids can scavenge reactive oxygen species (ROS), influence biological metabolic pathways, regulate gene expression, and thereby influence the resistance of plants to cold or frost stress. A study has shown that frost has an impact on the rhizosphere and thus also on the flavonoids in sorghum [31]. Sorghum exhibits a variation in flavonoid concentration depending on its genetic background. Frost can have an impact on the total concentration of flavonoids, which can decrease after frost stress, while the luteolinidin concentration can increase after frost [32]. In addition, it was also found that proline content in sorghum is accumulated under the influence of frost [24]. The effect of abscisic acid on freezing tolerance in rice was shown in a study [33], and a frost tolerance gene found in rice was also shown to improve frost tolerance in other crops [34, 35]. In maize, the influence of hormones and Ca2+-mediated signaling on the response to frost stress was investigated [30]. Prior acclimatization can increase frost tolerance, possibly due to changes in membrane lipid composition [24, 30]. Accordingly, sorghum exhibits greater tolerance to frost when previously exposed to cold temperatures [24].

Given the paucity of previous reports on tolerance sources for frost tolerance in sorghum and its genetic architecture, the aim of this study was to investigate a broad diversity set (*n* = 285) under semi-controlled short frost conditions, accompanied by scoring of emergence at early sowing under field conditions, to gain insights into the genetic architecture of these traits via GWAS. Since the interaction of these two traits is important for early sowing, we aimed to identify superior accessions that can be used to breed enhanced varieties suitable for Central European conditions.

## Materials & methods

### Plant material and analysis of population structure

A broad *S. bicolor* diversity set (*n* = 285 inbred lines) was utilized for this study. Aiming to cover a high diversity enabling high genetic variance and identification of relevant genomic regions for frost tolerance, this set includes all races (*S. bicolor*,* S. caudatum*,* S. durra*,* S. kafir and S. guinea*) and major geographical origins of sorghum. It comprises public photo-neutral conversion lines of globally diverse origins [36] on the one hand, and breeding lines developed and selected under German conditions (by a joint breeding program of Justus-Liebig-University Giessen, Norddeutsche Pflanzenzucht Hans-Georg Lembke KG and Deutsche Saatveredelung AG) on the other hand. The latter genotypes are adapted to local climate and soil conditions. Further, there is a high overlap (*n* = 159) with the diversity set utilized in [37] to analyze reproductive cold tolerance. The traits panicle harvest index (PHI), seed yield (SY) and seed number (SN) after reproductive cold stress presented therein are used here to compare the performance with the results for the survival of juvenile frost stress. Further, some European commercial sorghum varieties were included as checks. The detailed composition of the Diversity Set is shown in Supplementary T1.

The software STRUCTURE 2.3.4 [38] was used to analyze the population structure in the diversity set. Cluster numbers were detected using the method of [39], where *delta K* values show the best number for *K*-cluster as highest peak.

### Field trial (cold tolerance)

A field trial was carried out at the experimental station of the Justus-Liebig-University Giessen in Gross-Gerau, Germany (49° 55‘13.44 N 8° 29‘4.92 E) to observe the cold tolerance of the diversity set under early sowing field conditions. Due to low seed availability of some genotypes in the diversity set, only 264 genotypes could be tested in this trial. The seeds were produced in a winter nurserey in Mexico under optimal ripening conditions. Germination tests at warm temperature were performed on a randomly selected subset, showing high germination rates of > 94% for all samples.

The field trial location in the upper Rhine Valley provides a warm and sunny climate by central European standards along with a sandy soil which warms up quickly, offering good conditions for growing sorghum. However, due to the danger of night frost in late spring, sowing normally cannot take place until early May. To induce cold stress in this trial, the trial was sown on 8 April 2021, around four weeks earlier than the normal sorghum planting time at this location. An alpha lattice design with two replications was used. Entries were sown with a precision seeder at 3.5 cm seed spacing in single rows with 2.5 m length and 0.7 m row spacing. Unfortunately, due to technical errors at mechanical weed control, some genotypes were removed from the experiment, so that the statistical analysis was performed as RCBD eventually (since the original alpha-lattice design was compromised).

**Fig. 1:**
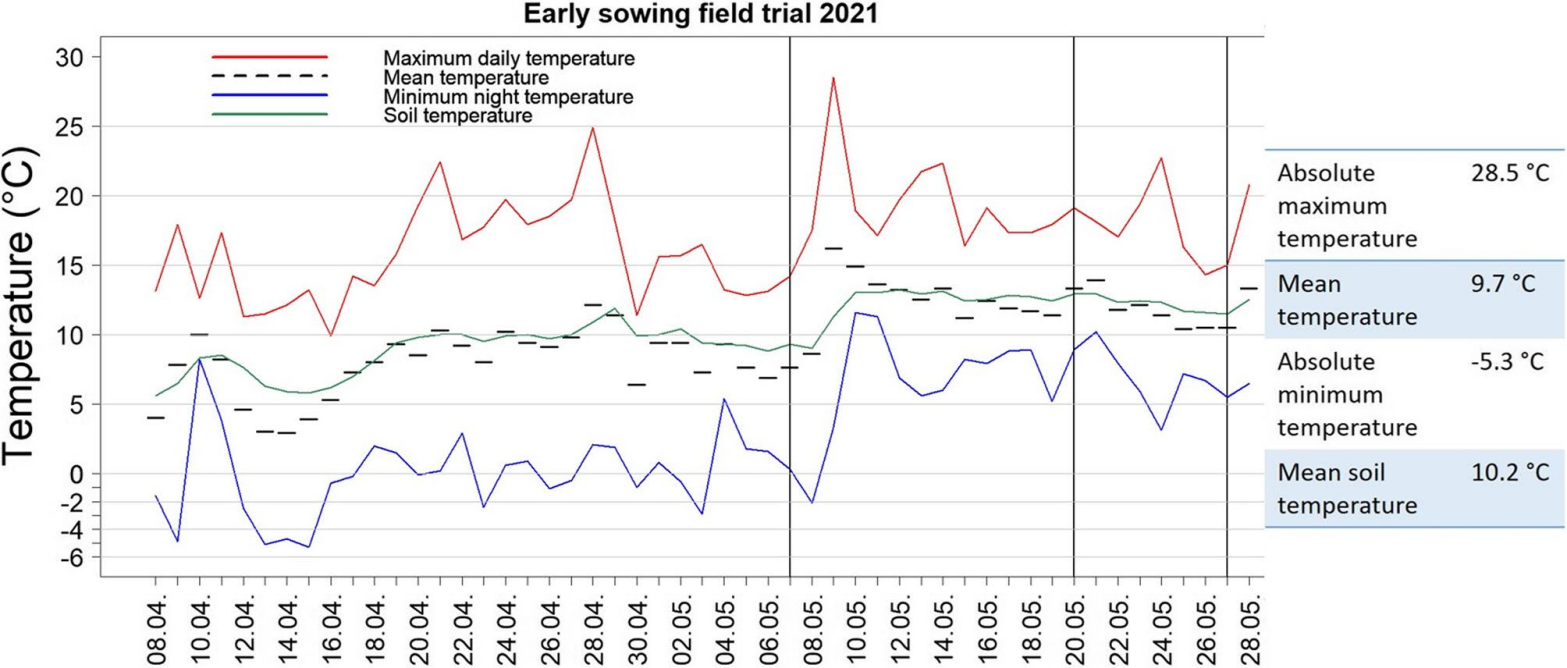
Maximum, minimum and soil temperature course during the early-sown field trial in Gross Gerau, Germany from April 8 (sowing date) to May 28, 2021. The vertical black lines indicate the dates on which plant emergence was counted. The temperature with absolute maximum (highest) and minimum (lowest) is shown in the box next to the figure

The number of emerged plants was counted three times (29, 42 and 50 days after sowing), after which no additional emergence was observed in the plots. When most of the plants were in 3-leaf-stage (BBCH 13) the trial was finalized and all entries were scored for early vigor on a scale of 1 (poor) to 9 (excellent). Temperature data during the trial were obtained by a weather station in Gross Gerau [40]. The temperature course during the field trial is shown in Fig. 1. The mean temperature, between 8 April and 28 May, 2021 was 9.7 °C, while the mean minimum temperature was 2.8 °C and the absolute minimum nighttime temperature (i.e. the lowest temperature during the course of the experiment) was − 5.3 °C. Sub-zero temperatures were recorded on a total of 16 nights. The mean maximum daytime temperature (red line) was 17 °C. Soil mean temperature (green line) was 10.2 °C and the minimum soil temperature was 5.6 °C. Counting dates are marked as a black vertical line (Fig. 1).

### Semi-controlled experiments (frost tolerance)

**Fig. 2:**
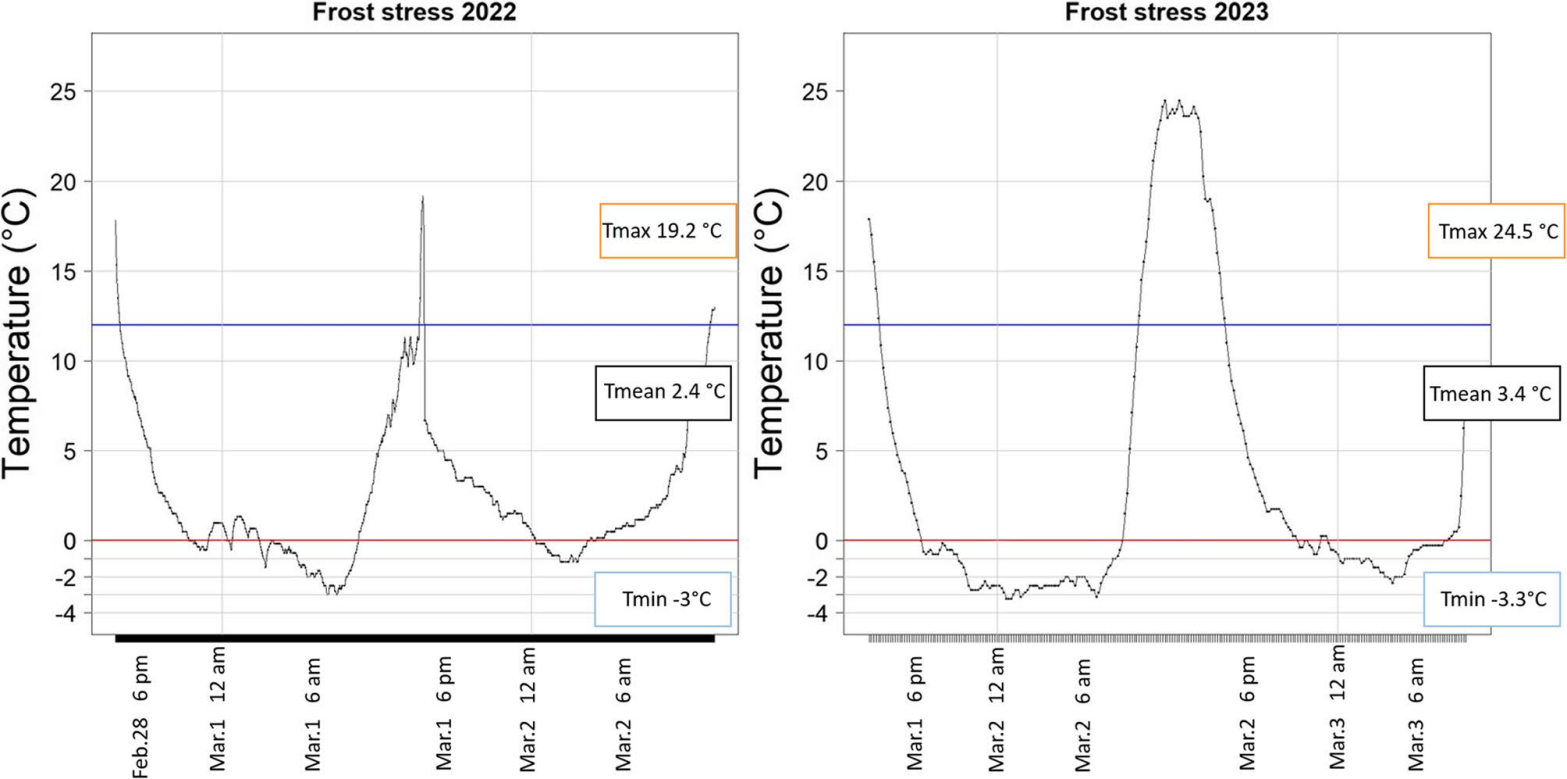
Temperature curve in °C according to date and time for the semi-controlled frost experiment in 2022 (left) and 2023 (right). The blue line indicates 15 °C threshold, being considered the threshold for cold stress in sorghum, and red line indicates 0 °C. Below the graphs, the absolute minimum and maximum temperature is shown. The temperature with absolute maximum (highest, Tmax), mean temperature (Tmean) and minimum (lowest, Tmin) is shown in the box next to the graph

In addition to the field trial, two independent semi-controlled experiments were conducted using the entire diversity set (*n* = 285) to investigate juvenile survival rate after frost stress. To simulate short frost periods in early May, which are very common in Germany (see Fig. 1), the plants were stressed by exposing them to freezing temperatures on two consecutive nights (three days) after their emergence under warm temperatures. These experiments were carried out in Giessen (Germany) from the end of February to the beginning of March in 2022 and 2023, respectively. Planting was done in cultivation plates with 96 pots (8 × 12 cm), with each pot containing 10 seeds. Sowing and subsequent emergence took place under controlled greenhouse conditions with temperatures of (day/night) 20 °C/16°C in 2022 and 18 °C/12°C in 2023, respectively. These controlled warm conditions prior to stress treatment were applied to generate uniform emergence and development for all genotypes, focusing on the survival rate after frost stress. Stress treatments were applied 14 days after sowing in 2022 and 16 days after sowing in 2023, when most plants reached the 3-leaf-stage (BBCH 13). The number of emerged plants in all pots was recorded before the stress treatment, for which all plants were transferred from the greenhouse chamber to an open space outdoors, where they were exposed to natural outdoor temperatures which dropped as low as −3 °C at night (Fig. 2). After the stress treatments, the plants were transferred back to the warm greenhouse chamber to facilitate recovery of stress-tolerant genotypes. After 10 days of recovery in controlled, warm conditions, the plants were scored for survival rate (number of living plants after stress/number of plants before stress) and visual stress symptoms (e.g. necrotic leaves) on a scale from 1 (death of plants) to 9 (no stress symptoms).

The first experiment in 2022 was performed in three replications using a completely randomized block design (RCBD). In 2023, the second experiment was performed as an alpha lattice design in four replications. During both experiments, the temperature was measured with three data loggers (EL-USB-2, Lascar Electronics, UK) which were placed between the juvenile plants on the ground level. Additional environmental conditions like wind, solar radiation and relative humidity can be found in the supplements (S-Figure 1). These data are taken from the weather station in Giessen, which is closest to the experiment.

In 2022, the average temperature during the stress period was 2.4 °C. During the first frost period, plants were exposed to temperatures below 0 °C for a cumulative period of around 450 min. During the second frost period, plants were exposed to temperatures below 0 °C for a cumulative period of around 200 min. The highest temperature across both periods was 19.2 °C and the lowest temperature was − 3 °C. In 2023, the mean temperature during the cold stress period was 3.4 °C. The first frost period had a total duration of around 840 min and the second frost period had a total duration of around 560 min. A maximum daily temperature of 24.5 °C was recorded across the two periods, with a minimum nighttime temperature of −3.3 °C (Fig. 2).

### Analysis of phenotypic variation

For the semi-controlled frost experiments, an analysis of variance (ANOVA) was performed for the traits survival and stress rating using the following generalized linear model, in which genotypes and environments (2022 and 2023) were considered as fixed and replicates as random effects:

1 $$\:\:\:{Y}_{ijk}\:\:\sim\:\mu\:\:+\:{g}_{i}\:+\:{e}_{j}\:+\:{\left(ge\right)}_{ij}\:+\:{R}_{k}\:+\:{e}_{ijk}$$

where Y_*ijk*_ is the observed dependent variable, µ represents the overall mean, g_*i*_ is the *i*th genotypic effect, e_*j*_ is the *j*th environmental effect, ge_*ij*_ is the genotype-by-environment interaction, R_*k*_ is the random effect of *k*th replication and e_*ijk*_ is the residual effect.

To compare the levels of genotypic variance obtained in the different years, ANOVA was also computed separately for each of both years using the following generalized linear model, where genotypes were considered as fixed and replicates as random effects:

2 $$\:{Y}_{ij}\:\:\sim\:\mu\:\:+\:{g}_{i}\:+\:{R}_{j}\:+\:{e}_{ij}$$

where Y_*ij*_ is the observed dependent variable, µ represents the overall mean, g_*i*_ is the *i*th genotypic effect, R_*j*_ is the random effect of *j*th replication and e_*ij*_ is the residual effect.

For the field trial, the same ANOVA formula [2] was used to calculate the variance for the considered traits emergence index, emergence in percent and vigor ranking.

The Emergence Index (EI) was calculated as described by [41]:

$$\:EI=\frac{[\sum\:\:({E}_{j}\:\times\:\:{D}_{j})]}{E}$$

where E_*j*_ is the number of newly emerged plants on day *j*, D_*j*_ is the day after planting, and E is the final seedling emergence (i.e. number of plants). Low emergence index values indicate a fast emergence, which is desired.

Emergence percentage (E (%) or Eper) was calculated according to the following formula:

$$\:E\:\left(\%\right)=\left(\frac{\text{T}\text{o}\text{t}\text{a}\text{l}\:\text{n}\text{u}\text{m}\text{b}\text{e}\text{r}\:\text{o}\text{f}\:\text{e}\text{m}\text{e}\text{r}\text{g}\text{e}\text{d}\:\text{s}\text{e}\text{e}\text{d}\text{s}}{\text{T}\text{o}\text{t}\text{a}\text{l}\:\text{n}\text{u}\text{m}\text{b}\text{e}\text{r}\:\text{o}\text{f}\:\text{p}\text{l}\text{a}\text{n}\text{t}\text{e}\text{d}\:\text{s}\text{e}\text{e}\text{d}\text{s}}\right)\:\times\:\:100$$

The coefficient of variation (CV) serves as a tool to compare the level of variation within the experiments. For the field trial and semi-controlled experiment the coefficient of variation was calculated as:

$$\:CV\:\left(\%\right)=\left(\frac{\text{S}\text{t}\text{a}\text{n}\text{d}\text{a}\text{r}\text{d}\:\text{d}\text{e}\text{v}\text{i}\text{a}\text{t}\text{i}\text{o}\text{n}}{\text{M}\text{e}\text{a}\text{n}\:\text{v}\text{a}\text{l}\text{u}\text{e}}\right)\:\times\:\:100$$

Pearson´s correlation coefficients were calculated for the traits emergence index, emergence in percent, vigor (field), survival and vigor (pot experiments 2022, 2023), panicle harvest index (PHI), seed yield (SY) and seed number (SN). For the latter three traits, data collected in in earlier experiments comprising different environmentswas used (see [37]).

All statistical analysis was performed using RStudio version 4.1.2 [42]. Plots were done by the R-package “ggplot2” [43] and the heatmap was created with R-package “metan” [44].

### SNP calling and GWAS

The genotype data of the diversity set contain 27,440 single-nucleotide polymorphism (SNP) markers. These marker data were obtained using DArTseq reduced-representation sequencing (DArT™, Canberra, Australia; www.diversityarrays.com) based on the *S. bicolor* reference genome BTx623 v3.1.1 (https://phytozome-next.jgi.doe.gov/info/Sbicolor_v3_1_1). For filtering the SNP data TASSEL 5.0 was used [45]. SNPs with more than 10% mssing data and a minor allele frequency of less than 5% wre removed. After filtering, a total of 6710 SNPs remained and were used for further analyses. The distribution of the SNPs across the genome is shown in the supplementary (S-Figure 2). To perform a genome-wide association study (GWAS), the R package *GAPIT* version 3 with a mixed linear model (MLM) was used [46, 47]. To control for environmental and experimental effects, the phenotypic values of the genotypes were estimated using BLUEs (Best Linear Unbiased Estimators) and these adjusted means over both years (2022 and 2023) were used for GWAS on the semi-controlled frost trial. To minimize type II errors, a significance threshold - log_10_ (p) ≥ 3 was used. Associated SNPs were compared to known QTL under cold stress for other traits from the Sorghum QTL Atlas [48] based on their physical positions. Phylogenetic relationships were calculated with TASSEL 5.0 using the neighbor-joining method [49]. The software Dendroscope 3.7.5 was used for visualization of the phylogram [50].

## Results

A highly significant genotypic variance (*p* ≤ 0.001) was found for all considered traits in all experiments, including emergence percentage and vigor in the field trial under early sowing conditions, as well as survival and vigor in the semi-controlled experiments with short frost periods. The trait Emergence Index showed only a significant genotypic variance (*p* ≤ 0.01).

### Field trial

The genotypes showed a wide range in terms of their emergence rate: while some emerged very quickly, other genotypes showed no emergence at all even at the end of the experiment. There is a significant difference in the emergence percentage, ranging from 0% (no emergence) to almost 50% of the seeds planted. The phenotypic differences between the genotypes in terms of their emergence capacity are confirmed by statistical analyses. The analysis of variance (ANOVA, Table 1) shows a highly significant variance (*p* < 0.001) among genotypes in terms of emergence percentage and vigor, as well as a significant variance (*p* < 0.01) in terms of emergence index (Table 1), which shows that the respective traits are influenced by different genetic backgrounds. All underlying data can be found in the supplementary Table T2.

**Table 1:** ANOVA and descriptive statistics for emergence index (EI), emergence percent (E (%) and Vigor scored during the early sown field trial in 2021 separated by genotype (G) and replication (Rep)

		EI					E (%)					Vigor				
	df	CV	MSq	Min	Mean	Max	CV	MSq	Min	Mean	Max	CV	MSq	Min	Mean	Max
G	263	20	49**	3.0	32.3	50.0	63	273***	0.0	18.8	48.5	49	2***	1.0	2.2	7.0
Rep	1		90.6					34.7					3.0*			
Error	263		34.5					78.2					0.7			

The coefficient of variation (CV) is added

The significance level are shown by ANOVA mean squares *p* ≤ 0.001***, *p* ≤ 0.01** and *p* ≤ 0.05*

### Semi-controlled experiment

**Fig. 3:**
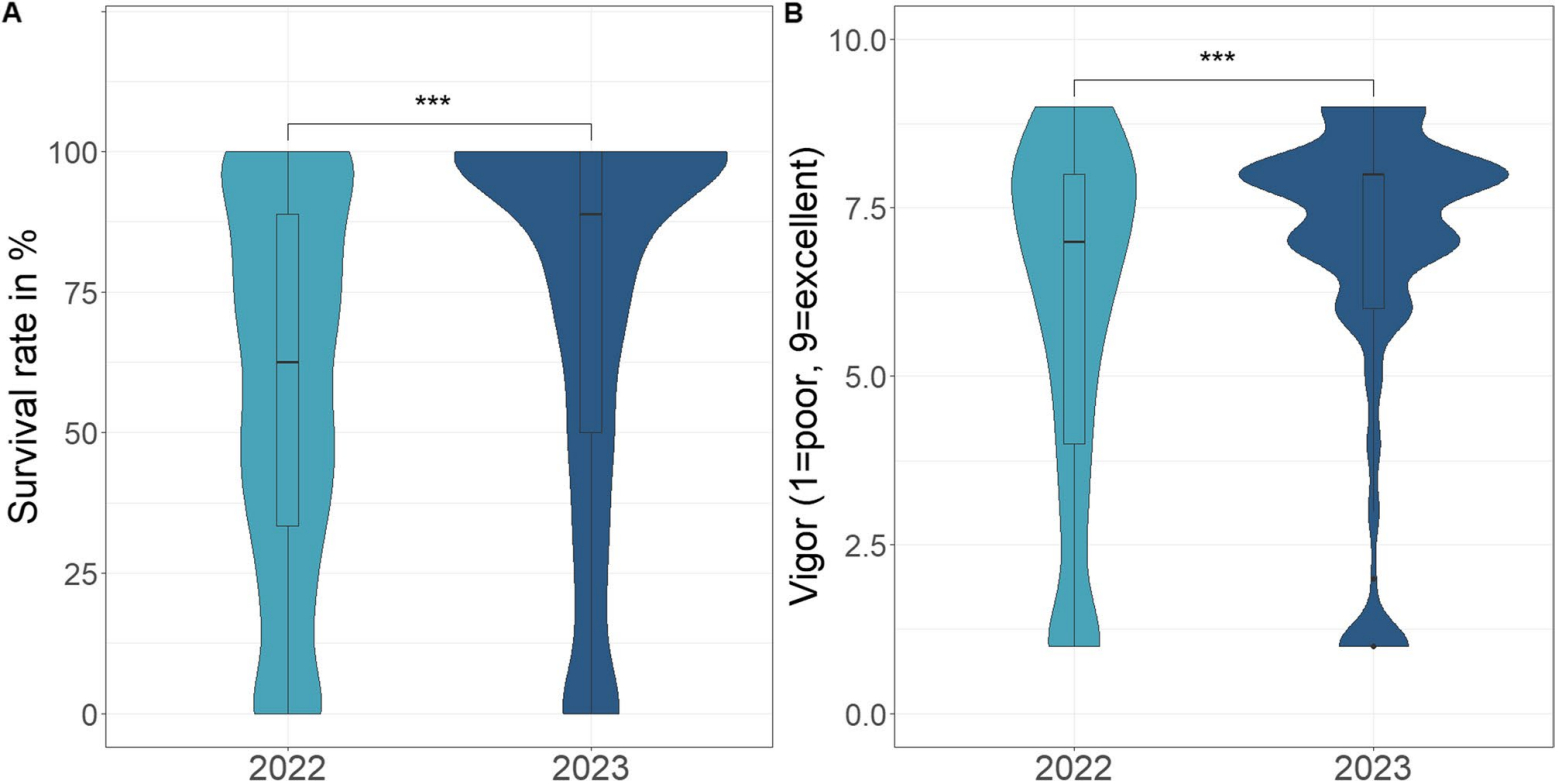
Violinplots for (**A**) percentage survival rate and (**B**) vigor for the two frost experiments, separately for the years 2022 and 2023. The significance level are shown by ANOVA mean squares *p* ≤ 0.001***, *p* ≤ 0.01** and *p* ≤ 0.05*

During the semi-controlled experiments, the entire range of survival rates was observed. This is evident from the fact that the survival rate in both experiments was between 0 and 100% and vigor values were between 1 and 9 (Fig. 3). Whereby the values for both characteristics, survival and vigor, are higher in 2023 than in 2022. The semi-controlled experiment in 2023 was designed as an alpha lattice design, but the analyses showed no block effects, so both experiments were treated as RCBD.

In both years, highly significant (*p* < 0.001) variances between genotypes were observed for the traits survival rate and vigor (Table 2), indicating a high influence of the genotypic background on trait performance. Furthermore, the interaction between genotypes and environmental conditions is highly significant (*p* < 0.001) and shows a higher survival rate and vigor in 2023 than in 2022 (Table 2; Fig. 3). All underlying data can be found in Supp. Table T3. For the data of 2023, the CV was approximately 46 for survival rate and 33 for vigor. In 2022, the survival rate had a higher CV of 56 compared to vigor with a CV of 43. This shows that the variation in appearance within the genotypes was lower than for the survival rate.

**Table 2:** ANOVA and descriptive statistics for survival rate (in %) and Vigor separated by genotype (G), environment (Env), genotype-environment interaction (G×E) and replication (Rep)

			Survival (%)		Vigor	
		df	CV	MeanSq	CV	MeanSq
2022 & 2023	G	284	51	2533***	38	11.32***
	Env	1		100,249***		299.90***
	G×E	284		1454***		6.44***
	Rep	3		3154**		26.08***
	Error	136		742		4.31
2022	G	284	56	2243***	43	10.3***
	Rep	2		7068***		112.4***
	Error	554		464		4.2
2023	G	284	46	1738***	33	7.49***
	Rep	3		3943**		8.96
	Error	805		915		4.19

The coefficient of variation (CV) is added. Results are shown separately for the individual years, as well as the pooled values for both years

The significance levels are shown by ANOVA mean squares *p* ≤ 0.001***, *p* ≤ 0.01** and *p* ≤ 0.05*

### Trait correlations

**Fig. 4:**
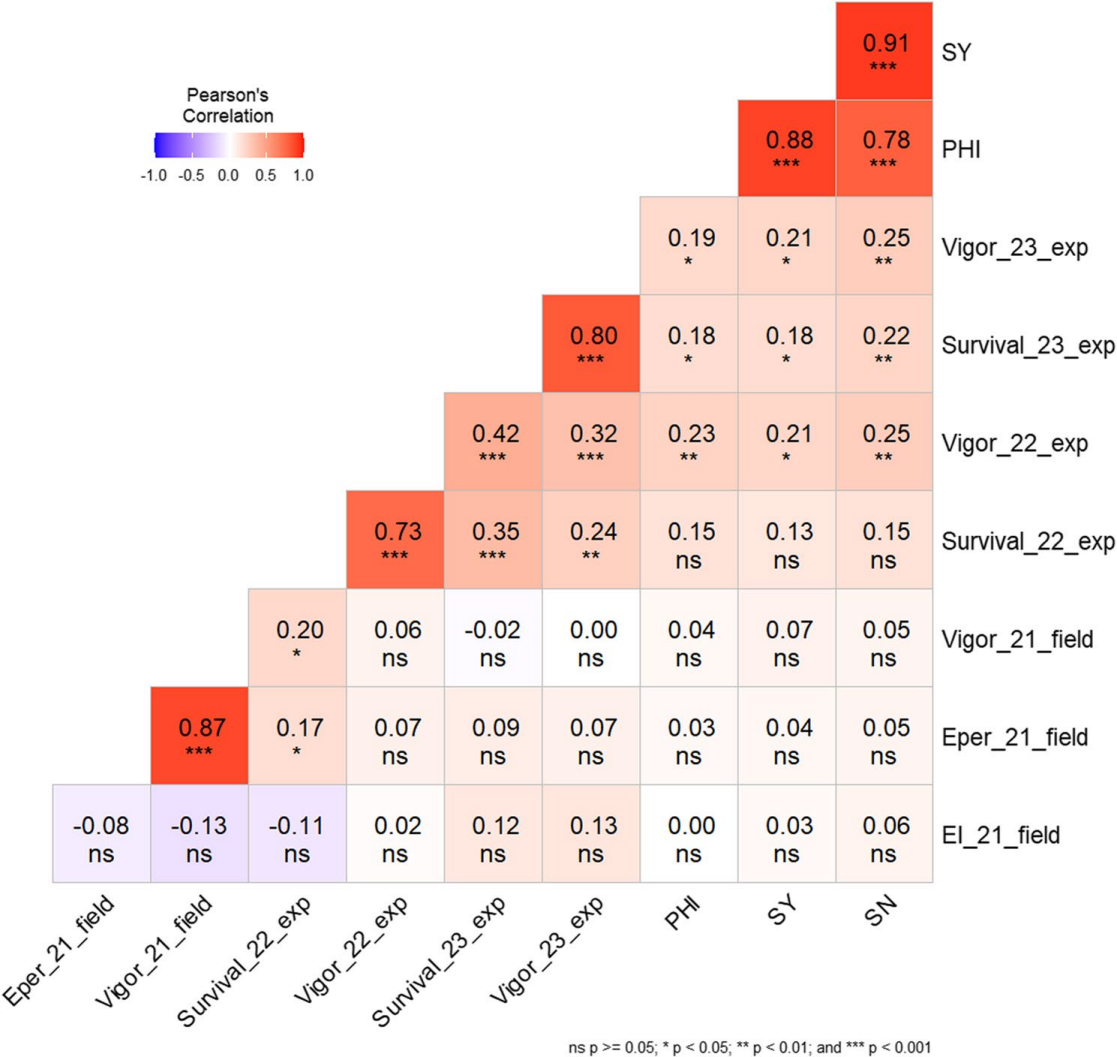
Pearson’s correlation for all scored traits Emergence Index (EI), emergence percent (Eper), vigor and survival (in %) from the field trial (2021) and semi-controlled frost experiments (2022 and 2023). The number following the trait abbreviation refers to the year. The reproductive cold tolerance traits panicle harvest index (PHI), seed yield (SY) and seed number (SN) were added from a previous study in other environments [37]

Pearson’s correlation coefficients were calculated among all considered traits of the present study (Fig. 4). For the semi-controlled experiments, a high correlation of *r* = 0.73*** (year 2022) and *r* = 0.80*** (year 2023) between survival rate and vigor was observed. Equally, in the field trial vigor showed a high correlation to the emergence percentage with *r* = 0.87***, but no correlation to the emergence index (*r* = 0.13). The correlation between the semi-controlled experiments of 2022 and 2023 was moderate for both vigor (*r* = 0.32***) and survival (*r* = 0.35***). Further, there was no correlation for vigor between field- and semi-controlled experiments. While emergence percentage to survival rate in 2022 was weakly correlated (*r* = 0.17*), no correlation (*r* = 0.09) could be found between field emergence in percentage to survival in 2023.

A high proportion of the lines (160 out of 285) tested in the present study for juvenile cold and frost tolerance had been scored for reproductive cold tolerance traits in previous trials [37]. To determine the correlation between juvenile and reproductive traits, the genotypic mean values of all four cold stress environments as described in Chakrabarty et al. for *panicle harvest index* (PHI), seed number (SN) and seed yield (SY) were considered. Weak correlations could be found between survival in both 2022 and 2023 and these reproductive cold tolerance traits. No correlation was found between yield performance after reproductive cold stress (PHI, SY and SN) and emergence (percentage and index) under early sowing conditions.

### Population structure

**Fig. 5:**
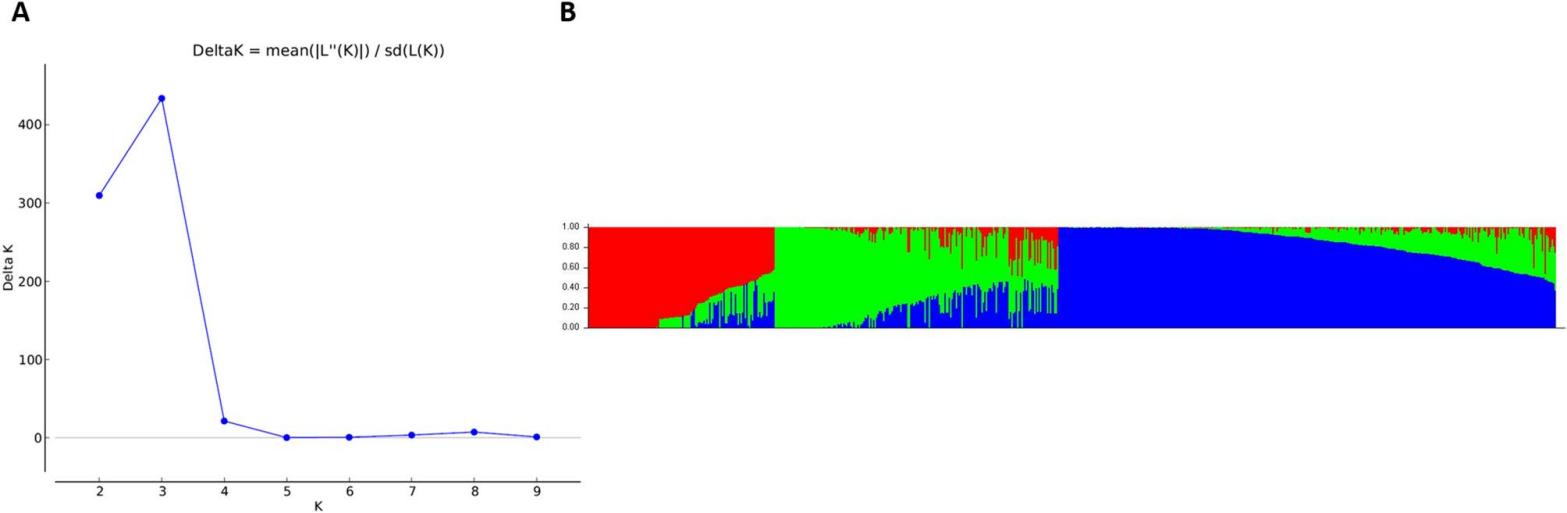
Classification of population structure with (**A**) estimation of cluster number with the best number of K = 3 as highest peak and (**B**) Barplot with three subpopulations shown as different colors

STRUCTURE analysis on the diversity set (*n* = 285) revealed grouping into three different subpopulations. Figure 5A shows the best estimated cluster number as *delta K* = 3. The barplot shows the distribution of the three clusters (Fig. 5B).

### Identification of superior genotypes

**Fig. 6:**
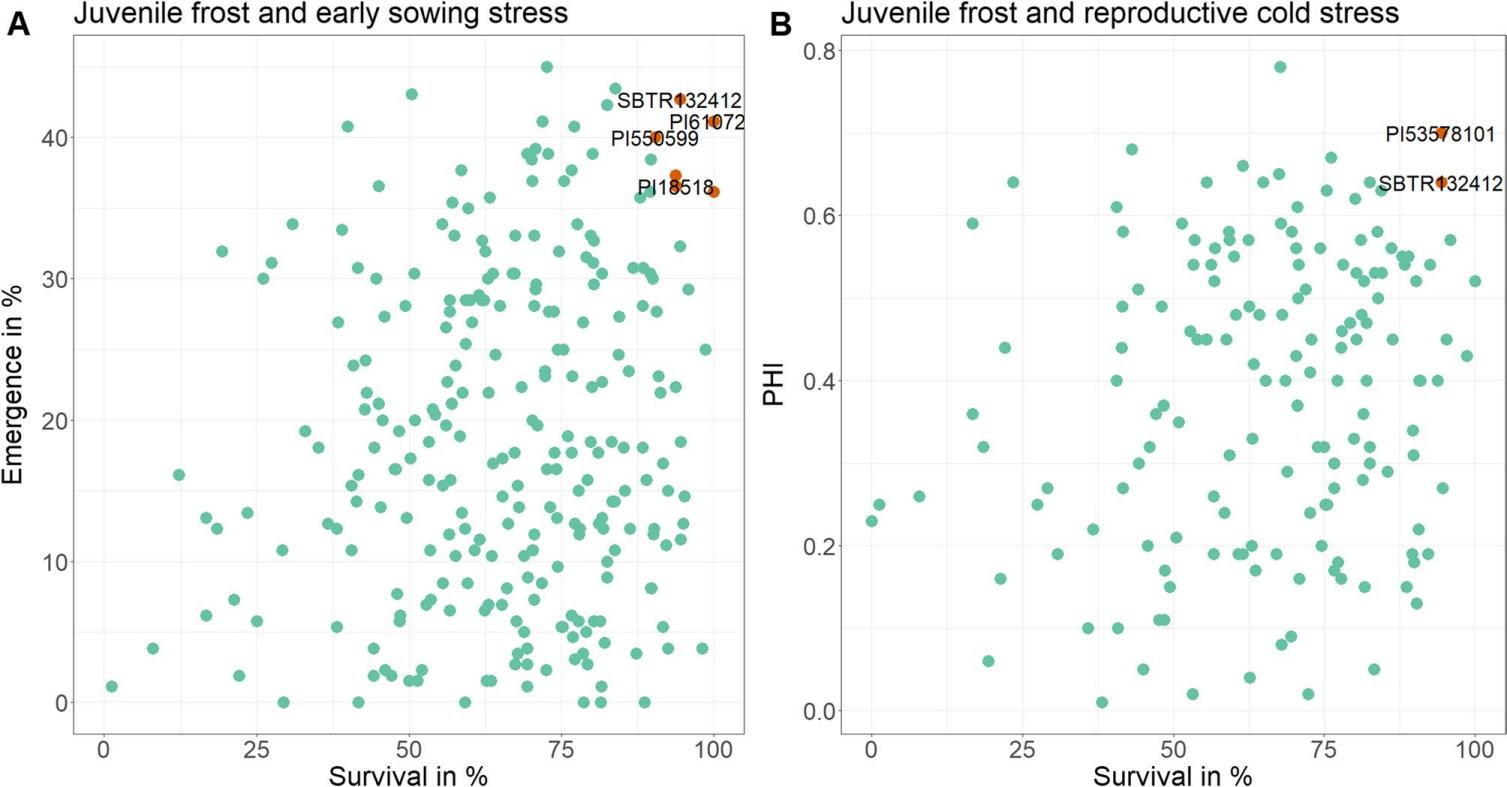
Scatterplot showing the best genotypes (**A**): emergence in percentage (above 35%) and survival rate (> 90%); (B) PHI (> 0.6) and survival rate (> 90%) after frost

High differences in the genotypic performance were observed. Some genotypes exhibited superior performance, with high emergence and survival rates in all three trials. Other genotypes displayed good emergence but were unable to survive the frost stress during the early stages. On the other hand, there were genotypes with low emergence but high survival rates after the frost stress. The survival rate here is shown as a mean of both frost experiments (2022 and 2023). Superior genotypes with a high survival rate (> 90%) and emergence in percent (> 35%) are shown as red dots in the top right-hand corner (Fig. 6 A). Two genotypes were found to have a high PHI (> 0.6) and survival rate (> 90%) (Fig. 6B). Just one genotype (SBTR132412) belongs to the superior with high performance in all juvenile and reproductive traits.

**Fig. 7:**
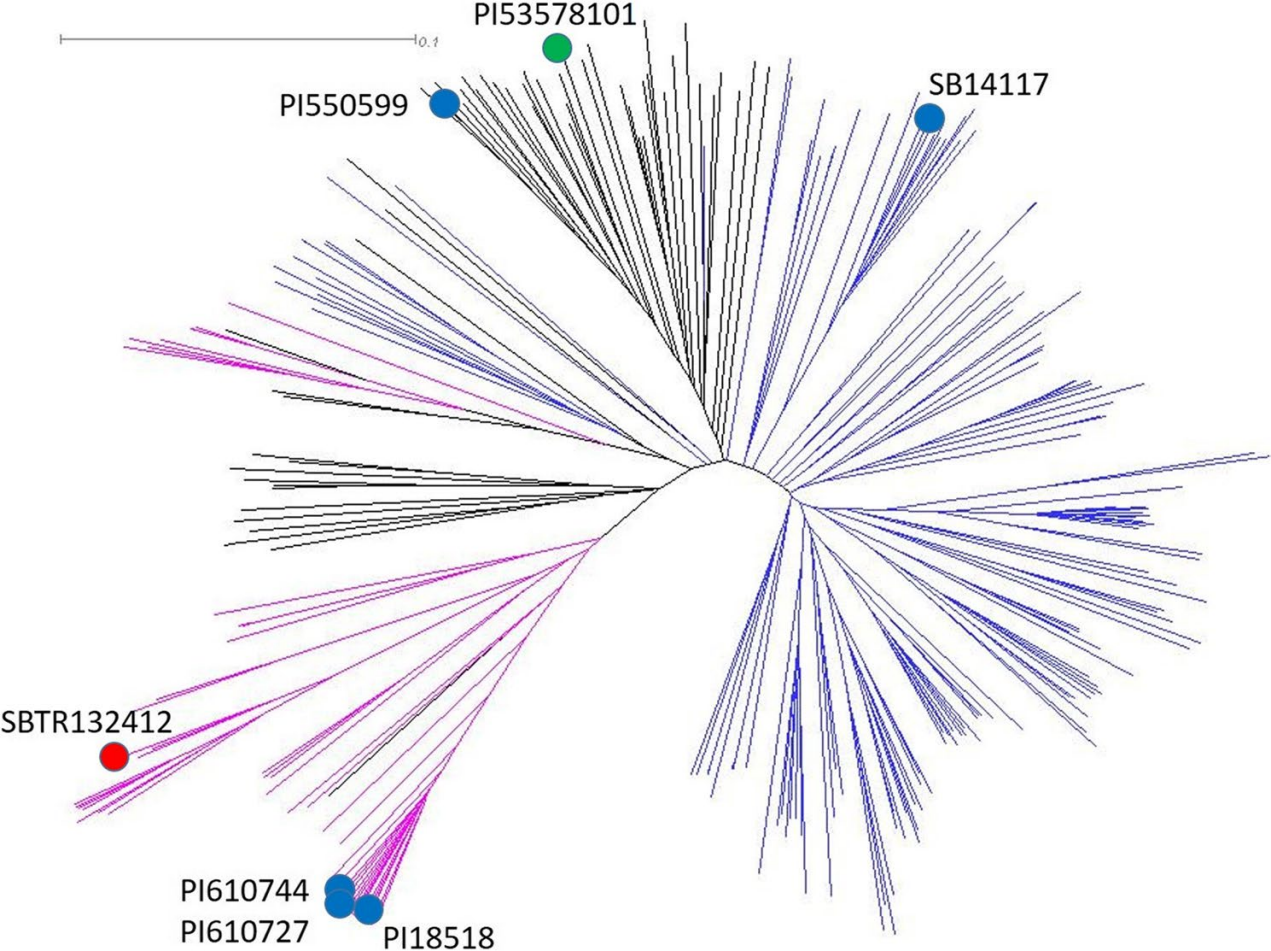
Phylogram for the sorghum diversity set (*n* = 285), showing classification of subpopulations according to the calculated clusters (blue, pink and black colored branches). Blue dots indicate superior genotypes with survival rate > 90% after frost and emergence > 35% under early-sown field conditions. The green dot shows one genotype found to have survival rate > 90% and PHI > 0.6 during reproductive cold stress. Red dot shows genotype SBTR132412, which had a high performance for all three traits

The diversity set was clustered into three subgroups (Fig. 5). These three groups are marked by different colors in the phylogram (Fig. 7). To present the genetic relatedness of these superior genotypes (Fig. 6), they are highlighted in the phylogram of the diversity set (Fig. 7). All three subpopulations include at least one superior genotype. As can be seen in the phylogram, these genotypes are not all directly related. Most of the superior genotypes (*n* = 4) are found in the pink cluster. Three genotypes are very closely related (PI610744, PI610727 and PI18518) and are all Chinese *kaoliang* accessions. The other genotype in this subpopulation (SBTR132412) is a breeding line from the Netherlands, standing out as the only genotype being among the best in all three traits. Genotype PI53578101, originating from the USA, shows a high PHI and survival rate. In contrast, genotype PI550599 in the same cluster (black colored) is of Russian origin. Breeding line SB14117 with origin in Germany is located in the blue colored cluster and among the best in emergence and survival.

### GWAS

**Fig. 8:**
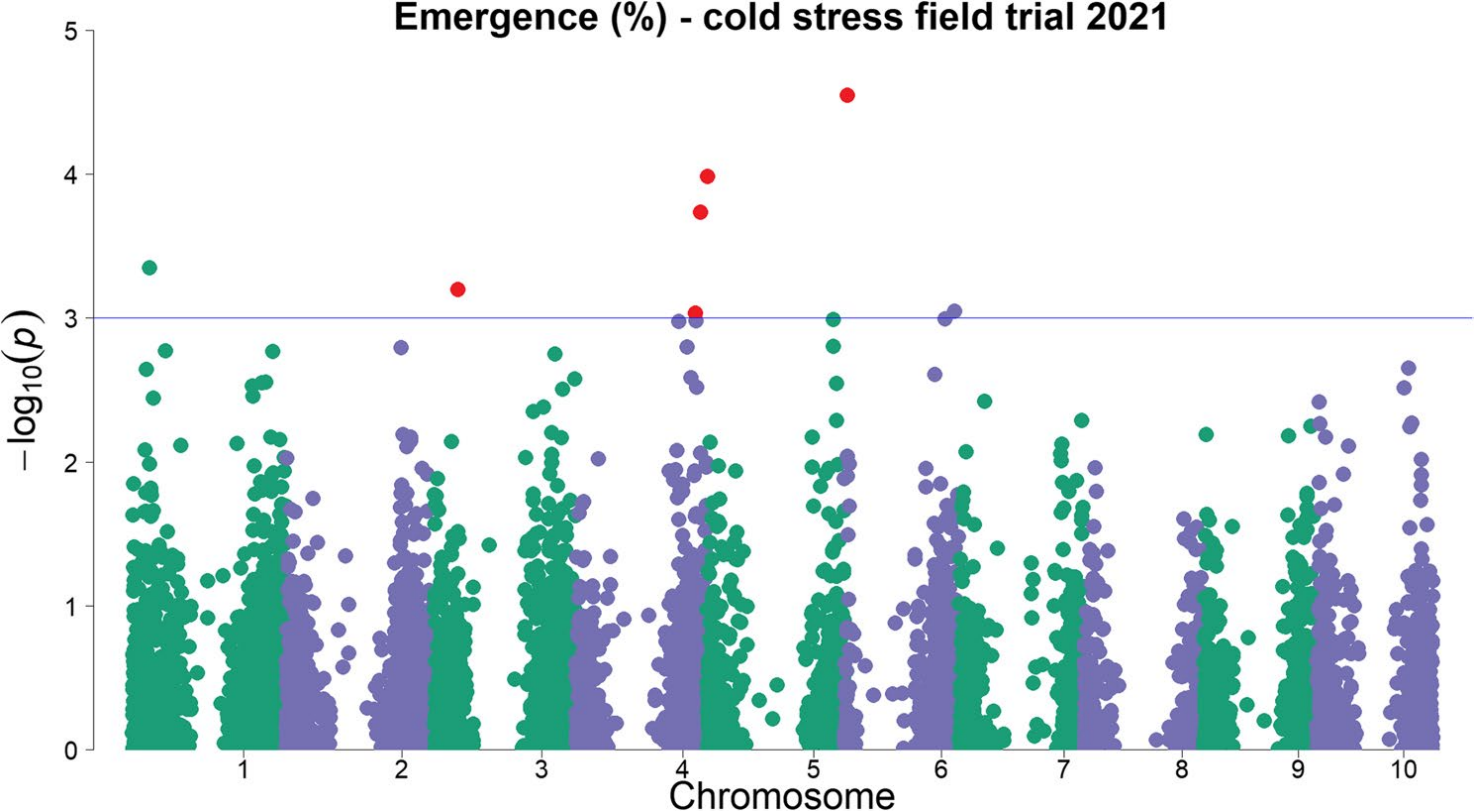
Manhattan plot of a genome-wide association study for the trait emergence in percent at early sowing. The chromosomes are shown on the x-axis and the negative decadic logarithm of the p-values on the y-axis. The higher the dots, the stronger is their association with the trait; the blue line shows the threshold of -log_10_ (p) ≥ 3. Individual dots represent genetic markers. The markers highlighted in red are significantly associated with the trait Emergence Index

A GWAS was carried out to identify genetic regions associated with the traits (emergence percentage, emergence index and survival rate), as already described in material and methods (2.5). The GWAS was performed separately for the different trials (cold stress and frost treatment, respectively). Several traits identified in this study were found to co-localize with previously known traits within the same genomic regions, as revealed by GWAS. Newly discovered associations involving survival were frequently located in regions also linked to physiological and developmental traits such as chlorophyll content, leaf development, and early growth [18, 51]. Similarly, emergence-related traits mapped to genomic regions already associated with emergence rate, growth performance, photosynthetic activity, and biomass-related traits [16, 18, 20, 51, 52]. These overlaps suggest a shared genetic basis or pleiotropic effects influencing both stress tolerance (e.g. survival under cold conditions) and early developmental processes (e.g. emergence and establishment). A full list of genomic regions associated to all traits is available in Supp. Table T4. Figure 8 shows a Manhattan plot representing the GWAS results with seven significantly associated markers for emergence percentage in the cold trial under field conditions with early sowing in 2021. Ten markers are associated with the trait emergence index. Figure 8 presents also the markers for the trait emergence index, which are shown as red markers. (A separate Manhattan plot for emergence index is not shown). In total 12 markers on chromosomes Sb01, Sb03, Sb04, Sb05 and Sb06 were found to be significantly associated with emergence percentage and emergence index under early sowing conditions.

**Fig. 9:**
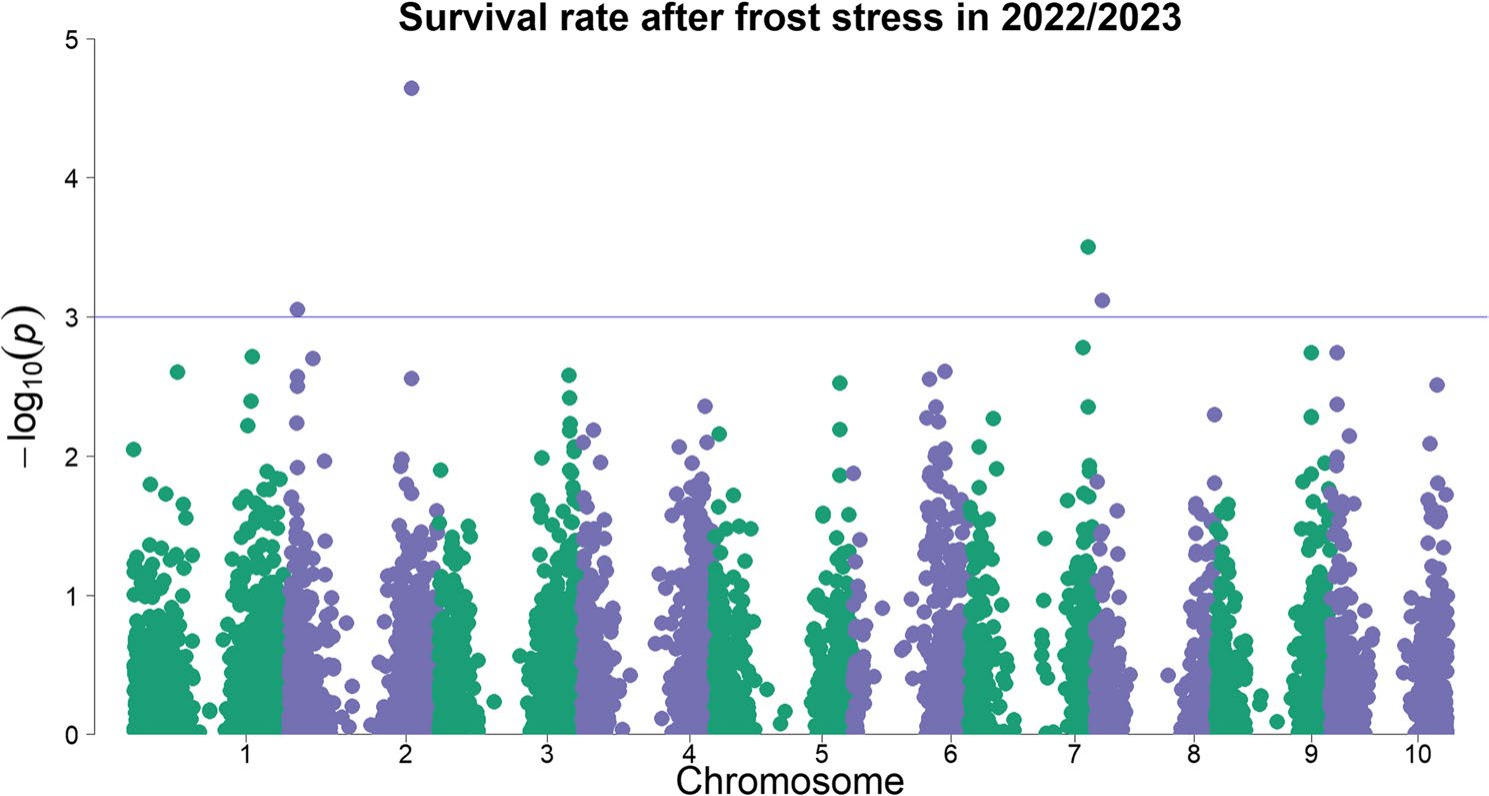
Manhattan plot of a genome-wide association study for the trait survival rate after short frost periods. The chromosomes are shown on the x-axis and the negative decadic logarithm of the p-values on the y-axis. The higher the dots are, the more they are associated with the trait; the blue line shows the threshold of -log_10_ (p) ≥ 3. Individual points represent genetic markers. The regions associated with the trait are located on chromosomes Sb02, Sb07 and Sb08

To control for environmental and experimental effects, the phenotypic values of the genotypes were estimated using BLUEs and these adjusted means over both years (2022 and 2023) were used for GWAS on the semi-controlled frost trial. Four SNP markers on chromosomes Sb02, Sb07 and Sb08 were significantly associated with plant survival after short frost periods (Fig. 9); however none of these markers overlap with GWAS peaks from the field trial. The markers on Sb02 are also highly associated with the trait survival rate according to GLM, MLMM, FarmCPU, and BLINK (results not shown). All associated markers were used to compared genome regions with known QTL in the Sorghum QTL Atlas, based on physical positions (genotype BTx623 used as reference genome for v3.0) of these markers with filtering criteria for cold tolerance and other abiotic stress tolerance (Supp. Table T5).

## Discussion

In this study different traits related to cold and frost tolerance in the juvenile stage in sorghum were analyzed. In the field trial, the aspect of emergence had an important impact under early sowing conditions with cold stress. To investigate the survival rate under short frost periods a semi-controlled greenhouse experiment was conducted. As the results show, there are big differences among the tested genotypes in response to cold and frost stress (Figs. 4 and 6).

### Potential for early sowing

The temperature during the field trial was well below the recommended temperature of 15 °C for sorghum [5, 6]. The poor emergence and delayed development underline the harsh conditions that were appropriate for the purpose of this trial to identify genetic differences (Fig. 1). However, for the present trial, it must be critically acknowledged that some of the plants died due to the cold during its course, which changes the ratio of the initial to the final stand and thus implies a rapid emergence. Whether a fast or slow emergence is advantageous depends on the ability of the individual genotypes to tolerate cold temperatures after emergence. Some genotypes show the same percentage emergence, but different emergence indices. The genotypes SC798 and PI542766 have an emergence around 30%, bu SC798 had a slow emergence and so a high emergence index, whereas PI542766 had a lower emergence index, because of plants which died during the course. Due to the lack of correlation between emergence index and emergence percentage, the emergence speed has no influence on the final stand in this trial (Fig. 4). Other studies showed negative correlations between these traits, whereas genotypes with a faster emergence showed a higher biomass [11, 13]. The lack of correlation between emergence index and emergence percentage (i.e. final number of established plants) despite significant genotypic differences in both traits suggests that emergence speed and field establishment are influenced by different genetic and physiological mechanisms. A genotype may emerge quickly but at the same time be sensitive to abiotic stress after germination, which has a negative impact on field establishment. Conversely, slower-germinating genotypes may have higher cold tolerance during autotrophic growth and show a better final establishment in consequence. Thus, the independence of both traits can be explained by the fact that rapid emergence under cold conditions does not necessarily correlate with better establishment in the field. Therefore, it is also important to look at other characteristics such as percent emergence, which represents the final field population. This trait correlates strongly with the vitality of the plants, which is assessed on the basis of appearance, including vigor and color (Fig. 4). The correlation between cold-tolerant genotypes has already been established [12, 13], indicating that the plants which germinate under cold conditions can also develop well, as photosynthesis does not appear to be impaired. However, in this experiment one can see that only about half of all the sown seeds emerged. This indicates that earlier sowing in cold soils is feasible, but it would require higher seed quantities to achieve the desired population density. This emphasizes the necessity of additional selection for cold germination and emergence.

### Sorghum can survive short Frost periods

To date, there has been limited research on frost tolerance of sorghum. It is important to note that cold tolerance and frost tolerance are not the same, as the physical properties of the plants are altered by freezing temperatures. This can be seen in the weak to non-existent correlation (Fig. 4) between emergence and survival. The results of the two frost trials (2022 and 2023) show relatively high survival rates, from which it can be concluded that sorghum can indeed survive short periods of frost (Fig. 3). This is also indicated by the strong correlation between survival rate and vigor (Fig. 4). In the study by [24], plants were almost completely killed after 24 h of frost stress at −3.5 °C, although the damage was slightly less if they were acclimatized beforehand. In the present study, it was shown that some genotypes could completely survive night temperatures down to −3 °C for a limited frost period. The duration of stress exposure has an effect on cold stress severity [53] and seems to have an effect on frost stress in comparison to the survival rate from [24] and the present study. The survival rate in 2023 was higher than in 2022, although the duration of night frost periods in 2023 was longer than in 2022 (Figs. 2 and 3), whereas the absolute minimum temperatures and average temperatures were almost the same. This contradicts the expectation that longer stress exposure leads to more damage, however survival is not only determined by stress duration but also by other environmental criteria such as wind and radiation. A noticeable difference between both years was a higher daytime temperature in 2023. Thus, even short periods with adequate temperature conditions for sorghum (above 15 °C) seem to reduce the degree of damage. However, some genotypes show large differences in survival rate between the two years, with only a moderate correlation being observed between the two frost trials. These genotypes show a higher genotype-by-environment interaction, which is already known from other cold tolerance studies [12, 13]. Overall, the magnitude of the G x E variance was around 57% of genotypic varince for survival. Other environmental influences such as solar radiation, wind and humidity can also have an impact on these differences. High solar radiation during a clear morning after a frost period is a typical scenario for late spring and likely to aggravate cell damage due to ROS [28].

### Genotypes with superior frost and cold tolerance

The fact that cold tolerance is not equivalent to frost tolerance [25] was underlined in our trials by the weak correlation between field emergence and frost survival. However, both traits are important for earlier sowing in Central Europe and some of the genotypes show high tolerance levels for both traits. These superior genotypes are important for further breeding programs. Since juvenile frost tolerance and reproductive cold tolerance correlate only weakly, it is difficult to find genotypes with good performance for both traits [37]. However, two genotypes could be found which belong to the top 10% of th survival rate after frost as well as to the top 10% underreproductive cold stress. These genotypes are likely to be the result of strong co-selection for both traits. Chinese genotypes have been identified as a source of cold tolerance and the three genotypes of Chinese origin in this study are cold tolerant *kaoliang* accessions [9, 22, 23]. The genotype PI610727 has been described as cold-tolerant in a previous study [6]. As can be seen in this study, these genotypes combine the ability to withstand short frost periods with a superior performance at above-freezing temperatures. The other three genotypes originate from the USA, Russia and Germany, respectively, suggesting that frost tolerance cannot be assigned to a specific geographical or phylogenetical region. One genotype (SBTR132412) of Dutch origin is even in the top 10% of all three traits indicating an efficient breeding program and the adaptation of this genotype to higher latitudes. As some cold tolerance genes are located close to plant height genes, these traits are commonly inherited together, which has led to a loss of cold tolerance due to long selection on short genotypes [23]. This seems to be also the case in the present study. All superior genotypes (Fig. 6A) have a plant height of more than 2 m, with the exception of genotype PI550599, which has a plant height of less than 1.5 m (plant height data not shown).

### Genome regions associated to cold and Frost tolerance

The trait “emergence in percent” is a quantitative trait as it is influenced by several genomic regions (Fig. 8). Previous studies have linked the significantly associated regions on chromosomes Sb01, Sb03, Sb04, Sb05 and Sb06 to cold tolerance. These studies examined emergence and vigor characteristics [12, 15, 16, 18, 20, 23, 51, 52, 54]. The co-localization of alleles for cold susceptibility with the desired alleles for zero-tannin (*tan*1) and short height (*dw*1 and *dw*3) has been postulated to have stymied breeding for enhanced cold tolerance in US grain sorghums [22, 23]. A co-localization (< 1 Mbp) of QTL for emergence percentage and emergence index with the major locus for tannin content (*Tan*1) on chromosome Sb04 was also detected in this study, but in contrast, no overlapping QTL with *Dw*1 or *Dw*3 [22, 23] were found here. Several QTL found in the present study on chromosomes Sb01, Sb04 and Sb05 for emergence percentage and emergence index are located close (< 5 Mbp) to QTL vigor and emergence detected in study [23]. This underlines the importance of the Sb04 chromosome for cold tolerance, which has already been shown in other early sowing trials [22]. The associated loci show that not all QTL for cold tolerance are related with undesirable traits and that it is therefore possible to select for tannin-free, short and yet cold-tolerant genotypes. The chromosomes containing QTL for plant survival after short frost periods do not appear to be associated with cold tolerance in this diversity set, suggesting that the two traits are under separate genetic control. This finding is not surprising, as the two traits are based on different physiological mechanisms and no phenotypic correlation was observed. In the study by [24], chromosomes Sb03, Sb04, Sb05 and Sb10 were found to be associated with the trait of frost tolerance. However, the study analyzed a recombinant inbred line (RIL) population, with consequently limited genetic diversity. In our present study, a large diversity set was evaluated, thus creating a broader genomic spectrum that may have caused some alleles to remain undetected. Overlapping with the regions identified on chromosome Sb02 for frost survival, previous studies have already demonstrated the importance of these regions under cold [18, 51]. The region on Sb02 is associated with chlorophyll content, which has an important influence on the survival of plants. This would go hand in hand with the strong correlation of surviving plants with vigor. No overlapping QTL with other abiotic stress tolerance traits were found in the QTL Atlas in the regions of chromosomes Sb07 and Sb08 where we detected QTL for frost tolerance. Out of the four QTL for frost survival found in the present study, two are close (< 5 Mbp) to gene loci for tannin content (*Tan*2, Sb02) and plant height (*Dw*3, Sb07). This finding coincides with the tall stature of most superior genotypes in this study. Therefore, comparable to cold tolerance [22, 23], frost tolerance has likely been lost during the selection of short, tannin-free genotypes. Nevertheless, since QTL for frost tolerance that are not related to height or tannins have also been found in the present study and [24], it may be possible to avoid undesirable traits during selection for frost tolerance. A very close QTL on Sb07 was found for plant survival, measured using the chlorophyll content under cold (but non-freezing) stress [18]. Under abiotic stress, there is an overlapping QTL in the same region of chromosome Sb07 for transpiration rate under drought stress conditions [55]. This could indicate that the region found under frost stress is not only associated with frost, but possibly also with other abiotic stress survival, which would confirm the nearby QTL for chlorophyll content by [18]. An overlapping QTL on Sb08 for salt tolerance under abiotic stress could be found [56]. In both salt and frost stress, the water balance is an important feature for preventing cell damage, with osmotically active compounds such as proline playing a role [24, 30, 57].

The identification of QTL associated with cold and frost tolerance in sorghum has relevant implications for breeding strategies. Marker-assisted selection (MAS) could facilitate the targeted introgression of favorable alleles into elite sorghum lines. This is especially useful in early selection stages, where genotyping can reduce the need for extensive phenotyping [58]. However, cold tolerance in sorghum is a complex, quantitative trait controlled by multiple small-effect loci, limiting the efficiency of MAS [59]. In this context, genomic selection (GS), which leverages genome-wide marker data to predict breeding values, offers a more powerful alternative for improving such traits [60]. Nevertheless, phenotypic selection remains relatively feasible in sorghum, as large populations can be evaluated under field conditions with low input costs. Therefore, the integration of genomic prediction and targeted phenotyping could represent the most efficient breeding approach for improving cold and frost tolerance in sorghum.

## Conclusions

In order to expand sorghum production further northwards into Central Europe, further enhancements in cold and frost tolerance during juvenile developmental phases are required. Considering this, the ability to emerge at cold temperatures under early sowing conditions and the survival of short frost periods in the juvenile phase are of great importance. Due to the extensive diversity examined in this study, a good overview of the suitability of the tested genotypes for early sowing was obtained. An important finding of this study was the high survival rate of numerous genotypes during short frost periods. The semi-controlled frost experiment under outdoor conditions proved to be a good testing option resembling actual field conditions, as all weather parameters affect the genotypes and can therefore influence the frost reaction. However, a relatively high amount of G x E interaction was observed across both experiments (2022 & 2023). Genome-wide association studies revealed genetic regions influencing frost survival. A combination of juvenile cold tolerance and frost tolerance was identified in superior genotypes, making them well suited for further breeding programs aiming at the establishment of sorghum in temperate latitudes. However, the finding that two out of four QTL for frost tolerance are located close to major loci for tannin content and plant height implies certain challenges for the breeding of frost tolerant, yet still short and tannin-free grain sorghums. Future research should address the question if this co-localisation implies pleiotropy or not, e.g. by gene-editing mediated knock-out of the well-known dominant alleles for tannins and tallness. Further research should tackle the validation of the identified QTL in different populations, and screening of the identified frost-tolerant accessions in broader environmental contexts, such as more field trials at stress locations and semi-controlled experiments with different temperature regimes.

## Supplementary Information

Supplementary Material 1.

Supplementary Material 2.

Supplementary Material 3.

## Data Availability

Phenotypic data are provided in supplements. All variants reported for the diversity set as reported by DArTseq are available at 10.5281/zenodo.11074146. The diversity set consist public conversion lines and genebank accessions purchased from USDA-ARS (https://npgsweb.ars-grin.gov/) and breeding lines from breeding program of Justus-Liebig-University Giessen. The classification of the genotypes can be found in the supplementary material with their description, origin and SC/PI number.

## Abbreviations

BLUE: Best Linear Unbiased Estimators
CV: Coefficient of variation
EI: Emergence Index
E (%)/Eper: Emergence percentage
GS: Genomic selection
GWAS: Genome-wide association study
G×E: genotype-environment interaction
MAS: Marker-assisted selection
PHI: Panicle harvest index
QTL: Quantative Trait Locus
RIL: Recombinant inbred line
SN: Seed number
SY: Seed yield

## References

[1] Awika JM. Major cereal grains production and use around the world: advances in cereal science: implications to food processing and health promotion. American Chemical Society; 2011.

[2] Poutanen KS, Kårlund AO, Gómez-Gallego C, Johansson DP, Scheers NM, Marklinder IM, et al. Grains - a major source of sustainable protein for health. Nutr Rev. 2022;80(6):1648–63. 34741520 10.1093/nutrit/nuab084 PMC9086769

[3] Kumar AA, Reddy BVS, Sharma HC, Hash CT, Rao PS, Ramaiah B, et al. Recent advances in Sorghum genetic enhancement research at ICRISAT. AJPS. 2011;02(04):589–600.

[4] Vadez V, Krishnamurthy L, Hash CT, Upadhyaya HD, Borrell AK. Yield, transpiration efficiency, and water-use variations and their interrelationships in the sorghum reference collection. Crop Pasture Sci. 2011;62(8):645.

[5] Yu J, Tuinstra MR. Genetic analysis of seedling growth under cold temperature stress in grain Sorghum. Crop Sci. 2001;41(5):1438–43.

[6] Burow G, Burke JJ, Xin Z, Franks CD. Genetic dissection of early-season cold tolerance in sorghum (Sorghum bicolor (L.) Moench). Mol Breed. 2011;28(3):391–402.

[7] Osuna-Ortega J, Mendoza-Castillo M, Mendoza-Onofre L. Sorghum cold tolerance, pollen production, and seed yield in the central high valleys of Mexico. Maydica. 2003;48:125–32.

[8] Maulana F, Tesso TT. Cold temperature episode at seedling and flowering stages reduces growth and yield components in Sorghum. Crop Sci. 2013;53(2):564–74.

[9] Cisse N, Ejeta G. Genetic variation and relationships among seedling Vigor traits in Sorghum. Crop Sci. 2003;43(3):824–8.

[10] Salas Fernandez MG, Schoenbaum GR, Goggi AS. Novel germplasm and screening methods for early cold tolerance in Sorghum. Crop Sci. 2014;54(6):2631–8.

[11] Yu J, Tuinstra M, Claassen M, Gordon W, Witt M. Analysis of cold tolerance in sorghum under controlled environment conditions. Field Crops Res. 2004;85(1):21–30.

[12] Schaffasz W, Friedt, Snowdon W. Sorghum as a novel crop for central europe: using a broad diversity set to dissect Temperate-Adaptation. Agronomy. 2019;9(9):535.

[13] Rutayisire A, Lubadde G, Mukayiranga A, Edema R. Response of Sorghum to Cold Stress at Early Developmental Stage. International Journal of Agronomy. 2021;2021:1–10.

[14] Vera Hernández PF, Mendoza Onofre LE. Rosas Cárdenas fdf. Responses of sorghum to cold stress: A review focused on molecular breeding. Front Plant Sci. 2023;14:1124335. 36909409 10.3389/fpls.2023.1124335 PMC9996117

[15] Fiedler K, Bekele WA, Friedt W, Snowdon R, Stützel H, Zacharias A, et al. Genetic dissection of the temperature dependent emergence processes in sorghum using a cumulative emergence model and stability parameters. Theor Appl Genet. 2012;125(8):1647–61. 22847026 10.1007/s00122-012-1941-4

[16] Ortiz D, Hu J, Salas Fernandez MG. Genetic architecture of photosynthesis in Sorghum bicolor under non-stress and cold stress conditions. J Exp Bot. 2017;68(16):4545–57. 28981780 10.1093/jxb/erx276 PMC5853419

[17] Marla SR, Shiva S, Welti R, Liu S, Burke JJ, Morris GP. Comparative transcriptome and lipidome analyses reveal molecular chilling responses in chilling-Tolerant sorghums. Plant Genome 2017; 10(3). 10.3835/plantgenome2017.03.0025 29293808

[18] Bekele WA, Fiedler K, Shiringani A, Schnaubelt D, Windpassinger S, Uptmoor R, et al. Unravelling the genetic complexity of sorghum seedling development under low-temperature conditions. Plant Cell Environ. 2014;37(3):707–23. 24033406 10.1111/pce.12189

[19] Windpassinger S, Friedt W, Deppé I, Werner C, Snowdon R, Wittkop B. Towards enhancement of Early-Stage chilling tolerance and root development in Sorghum F1 hybrids. J Agron Crop Sci. 2017;203(2):146–60.

[20] Knoll J, Gunaratna N, Ejeta G. QTL analysis of early-season cold tolerance in sorghum. Theor Appl Genet. 2008;116(4):577–87. 18097644 10.1007/s00122-007-0692-0

[21] Chakrabarty S, Mufumbo R, Windpassinger S, Jordan D, Mace E, Snowdon RJ, et al. Genetic and genomic diversity in the sorghum gene bank collection of Uganda. BMC Plant Biol. 2022;22(1):378. 35906543 10.1186/s12870-022-03770-y PMC9335971

[22] Marla S, Felderhoff T, Hayes C, Perumal R, Wang X, Poland J et al. Genomics and phenomics enabled prebreeding improved early-season chilling tolerance in Sorghum. G3 (Bethesda) 2023; 13(8). 10.1093/g3journal/jkad116 PMC10411554 37232400

[23] Marla SR, Burow G, Chopra R, Hayes C, Olatoye MO, Felderhoff T et al. Genetic architecture of chilling tolerance in Sorghum dissected with a nested association mapping population. G3 (Bethesda) 2019; 9(12):4045–57. 10.1534/g3.119.400353 PMC6893202 31611346

[24] La Borde N, Dweikat I. Identification of genomic regions associated with seedling Frost tolerance in Sorghum. Genes (Basel) 2023; 14(12). 10.3390/genes14122117 PMC10743030 38136939

[25] Palta and Weiss, editor. Ice for mation and freezing injury: an overview on the survival mechanisms and molecular aspects of injury and cold acclimation in herbaceous plants; 2018.

[26] Suzuki N, Mittler R. Reactive oxygen species and temperature stresses: A delicate balance between signaling and destruction. Physiol Plant. 2006;126(1):45–51.

[27] Ben Rejeb I, Pastor V, Mauch-Mani B. Plant responses to simultaneous biotic and abiotic stress: molecular mechanisms. Plants (Basel). 2014;3(4):458–75. 27135514 10.3390/plants3040458 PMC4844285

[28] Asada K. Production and scavenging of reactive oxygen species in chloroplasts and their functions. Plant Physiol. 2006;141(2):391–6. 16760493 10.1104/pp.106.082040 PMC1475469

[29] Mansour MMF, Emam MM, Salama KHA, Morsy AA. Sorghum under saline conditions: responses, tolerance mechanisms, and management strategies. Planta. 2021;254(2):24. 34224010 10.1007/s00425-021-03671-8

[30] Thomashow M. Role of Cold-Responsive genes in plant freezing tolerance. Plant Physiol. 1998;118:1–8. 9733520 10.1104/pp.118.1.1 PMC1539187

[31] Li J, Yu Q, Liu C, Zhang N, Xu W. Flavonoids as key players in cold tolerance: molecular insights and applications in horticultural crops. Hortic Res 2025;12(4). 10.1093/hr/uhae366 PMC11894532 40070400

[32] Cloutier M, Chatterjee D, Elango D, Cui J, Bruns MA, Chopra S. Sorghum root flavonoid chemistry, cultivar, and Frost stress effects on rhizosphere Bacteria and Fungi. Phytobiomes J. 2021;5(1):39–50.

[33] Shinkawa R, Morishita A, Amikura K, Machida R, Murakawa H, Kuchitsu K et al. Abscisic acid induced freezing tolerance in chilling-sensitive suspension cultures and seedlings of rice. BMC Res Notes 2013;6(351). 10.1186/1756-0500-6-351 PMC3766701 24004611

[34] Vannini C, Locatelli F, Bracale M, Magnani E, Marsoni M, Osnato M, et al. Overexpression of the rice Osmyb4 gene increases chilling and freezing tolerance of Arabidopsis thaliana plants. Plant J. 2004;37(1):115–27. 14675437 10.1046/j.1365-313x.2003.01938.x

[35] Soltész A, Vágújfalvi A, Rizza F, Kerepesi I, Galiba G, Cattivelli L, et al. The rice Osmyb4 gene enhances tolerance to Frost and improves germination under unfavourable conditions in Transgenic barley plants. J Appl Genet. 2012;53(2):133–43. 22246661 10.1007/s13353-011-0081-x

[36] Stephens JC, Miller FR, Rosenow DT. Conversion of alien sorghums to early combine genotypes. Crop Sci. 1967;7:396.

[37] Chakrabarty S, Kravcov N, Schaffasz A, Snowdon RJ, Wittkop B, Windpassinger S. Genetic architecture of novel sources for reproductive cold tolerance in Sorghum. Front Plant Sci. 2021;12:772177. 34899798 10.3389/fpls.2021.772177 PMC8652046

[38] Earl DA, vonHoldt BM. STRUCTURE HARVESTER: a website and program for visualizing STRUCTURE output and implementing the Evanno method. Conserv Genet Resour. 2012;4(2):359–61.

[39] Evanno G, Regnaut S, Goudet J. Detecting the number of clusters of individuals using the software STRUCTURE: a simulation study. Mol Ecol. 2005;14(8):2611–20. 15969739 10.1111/j.1365-294X.2005.02553.x

[40] LLH Agrarmeteorologie Hessen. Agrarmeteorologie Hessen [cited 2024 Feb 29]. Available from URL: www.wetter.llh-hessen.de/.

[41] Smith PG, Millet AH. Germinating and sprouting responses of the tomato at low temperatures. J Am Soc Hortic Sci. 1964;84:480–4.

[42] R Core Team. R: A language and environment for statistical computing. 2021. Available from: URL: https://www.R-project.org/.

[43] Wickham H. ggplot2: Elegant Graphics for Data Analysis; 2016.

[44] Olivoto T, Lúcio AD. Metan: an R package for multi-environment trial analysis. Methods Ecol Evol. 2020;11(6):783–9.

[45] Bradbury PJ, Zhang Z, Kroon DE, Casstevens TM, Ramdoss Y, Buckler ES. TASSEL: software for association mapping of complex traits in diverse samples. Bioinformatics. 2007;23(19):2633–5. 17586829 10.1093/bioinformatics/btm308

[46] Zhang Z, Ersoz E, Lai C-Q, Todhunter RJ, Tiwari HK, Gore MA, et al. Mixed linear model approach adapted for genome-wide association studies. Nat Genet. 2010;42(4):355–60. 20208535 10.1038/ng.546 PMC2931336

[47] Lipka AE, Tian F, Wang Q, Peiffer J, Li M, Bradbury PJ, et al. GAPIT: genome association and prediction integrated tool. Bioinformatics. 2012;28(18):2397–9. 22796960 10.1093/bioinformatics/bts444

[48] Mace ES, Innes D, Hunt C, Wang X, Tao Y, Baxter J, Hassall M, Hathorn A, Jordan DR. The Sorghum QTL atlas: a powerful tool for trait dissection, comparative genomics and crop improvement. Theoretical and Applied Genetics; 2018. 10.1007/s00122-018-3212-5 30343386

[49] Saitou N, Nei M. The neighbor-joining method a new method for reconstructing phylogenetic trees. Mol Biol Evol. 1987;4:406–25. 3447015 10.1093/oxfordjournals.molbev.a040454

[50] Huson DH, Richter DC, Rausch C, Dezulian T, Franz M, Rupp R. Dendroscope: an interactive viewer for large phylogenetic trees. BMC Bioinformatics. 2007;8:460. 18034891 10.1186/1471-2105-8-460 PMC2216043

[51] Fiedler K, Bekele WA, Matschegewski C, Snowdon R, Wieckhorst S, Zacharias A, et al. Cold tolerance during juvenile development in sorghum: a comparative analysis by genomewide association and linkage mapping. Plant Breeding. 2016;135(5):598–606.

[52] Fiedler K, Bekele WA, Duensing R, Gründig S, Snowdon R, Stützel H, et al. Genetic dissection of temperature-dependent sorghum growth during juvenile development. Theor Appl Genet. 2014;127(9):1935–48. 25023408 10.1007/s00122-014-2350-7

[53] Ercoli L, Mariotti M, Masoni A, Arduini I. Growth responses of sorghum plants to chilling temperature and duration of exposure. Eur J Agron. 2004;21(1):93–103.

[54] Moghimi N. Moghimi 2019 New candidate loci and marker genes on chromosome 7 for improved chilling tolerance. 10.1093/jxb/erz143 30949711

[55] Kapanigowda MH, Payne WA, Rooney WL, Mullet JE, Balota M. Quantitative trait locus mapping of the transpiration ratio related to preflowering drought tolerance in sorghum (Sorghum bicolor). Funct Plant Biol. 2014;41(11):1049–65. 32481057 10.1071/FP13363

[56] Wang H, Chen G, Zhang H, Liu B, Yang Y, Qin L, et al. Identification of QTLs for salt tolerance at germination and seedling stage of Sorghum bicolor L. Moench Euphytica. 2014;196(1):117–27.

[57] Paul M, Hasegawa RA, Bressan J-K, Zhu, Bohnert HJ. Plant cellular and molecular responses to high salinity. 10.1146/annurev.arplant.51.1.463 15012199

[58] Collard BCY, Mackill DJ. Marker-assisted selection: an approach for precision plant breeding in the twenty-first century. Philos Trans R Soc Lond B Biol Sci. 2008;363(1491):557–72. 17715053 10.1098/rstb.2007.2170 PMC2610170

[59] Bernardo R. Molecular markers and selection for complex traits in plants: learning from the last 20 years. Crop Sci. 2008;48(5):1649–64.

[60] Heffner EL, Sorrells ME, Jannink J-L. Genomic selection for crop improvement. Crop Sci. 2009;49(1):1–12.

