# Supplementary figures and images for: Genetic diversity and genome-wide associations for frost survival in sorghum
Source: BMC Plant Biol. 2025 Jul 28;25:966. doi: 10.1186/s12870-025-07014-7 (PMC12302733; doi:10.1186/s12870-025-07014-7)

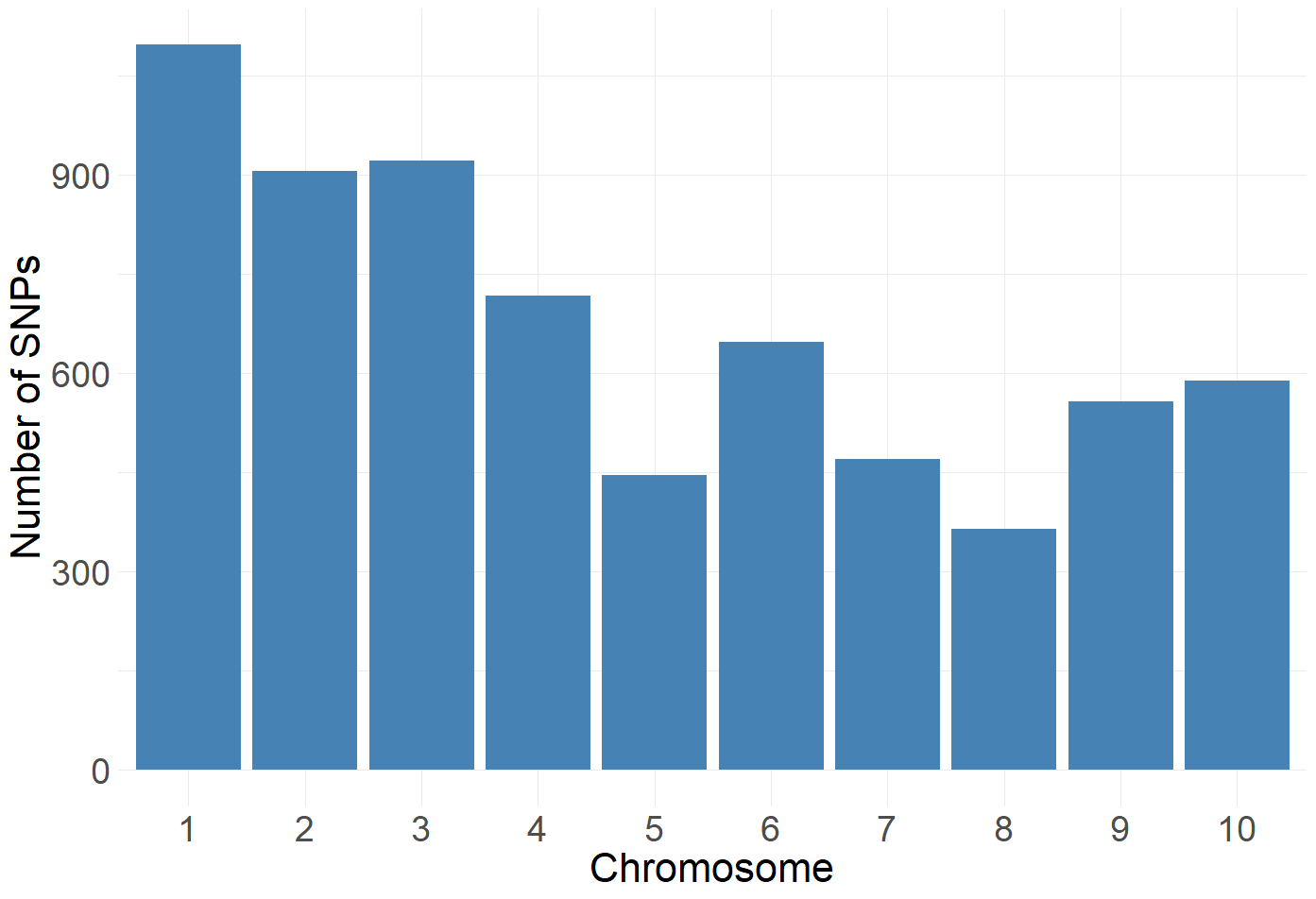

Supplement: Supplementary file 1 — Supplementary Material 1. [file 12870_2025_7014_MOESM1_ESM.png]

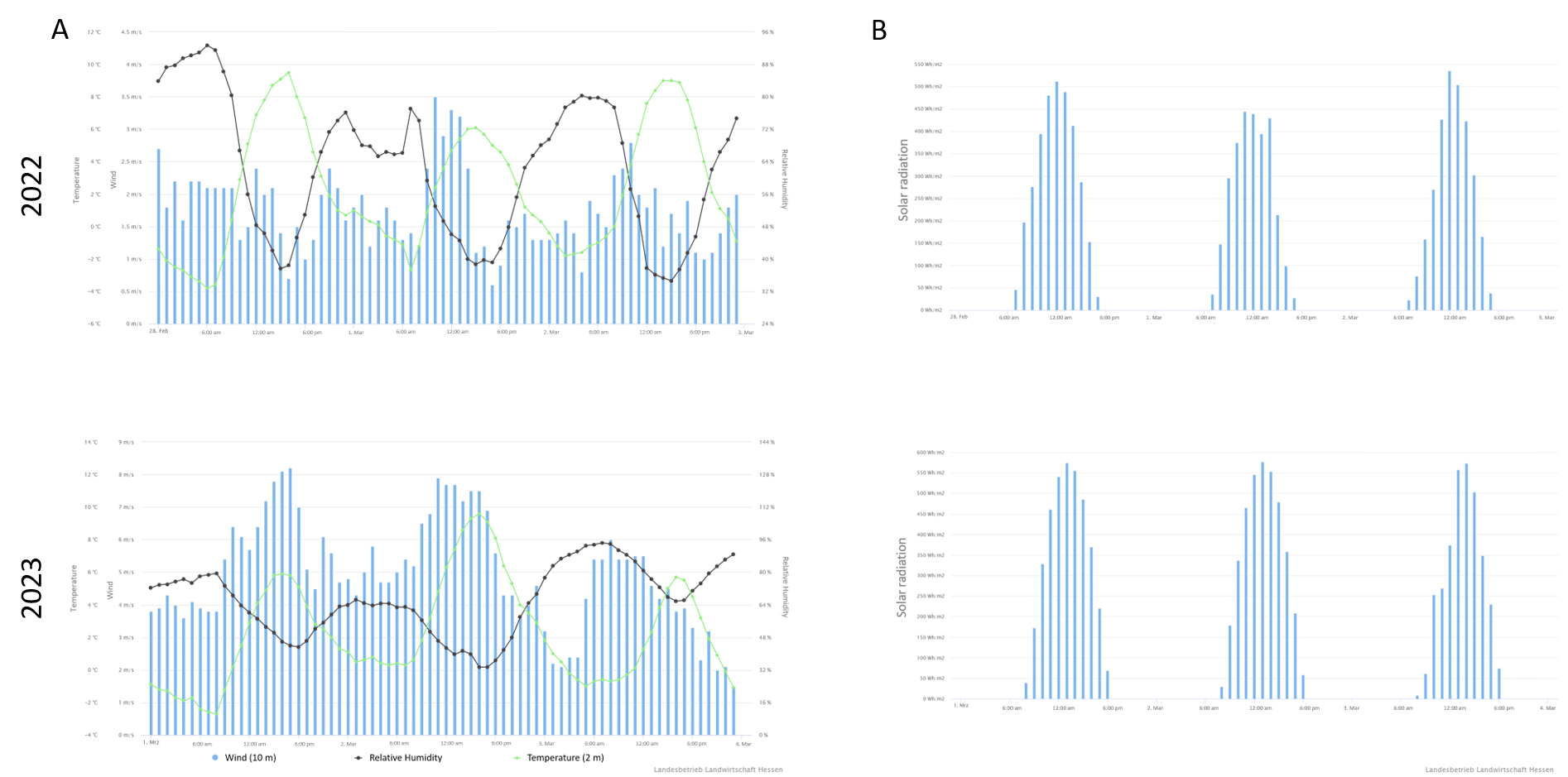

Supplement: Supplementary file 2 — Supplementary Material 2. [file 12870_2025_7014_MOESM2_ESM.png]
